# Prognostic value of the CT-based Suidan scoring system in advanced epithelial ovarian cancer

**DOI:** 10.3389/fonc.2026.1777388

**Published:** 2026-04-07

**Authors:** Chao Deng, Dan Yan, Hai-Bo Qu, Yan Zeng, Li-Hua Deng, Feng-Lin Jia, Xi-Jian Chen, Gang Ning

**Affiliations:** 1 Department of Radiology, West China Second University Hospital, Sichuan University, Key Laboratory of Birth Defects and Related Diseases of Women and Children of Ministry of Education, Chengdu, Sichuan, China; 2 Department of Radiology, The First People’s Hospital of Neijiang, Neijiang, Sichuan, China

**Keywords:** advanced epithelial ovarian cancer, CT imaging, prognosis, Suidan score, treatment strategies

## Abstract

**Purpose:**

This study aimed to evaluate the prognostic utility of the Suidan CT-based scoring system in patients with advanced epithelial ovarian cancer (AEOC) undergoing either primary debulking surgery (PDS) or neoadjuvant chemotherapy followed by interval debulking surgery (NACT-IDS). The score’s correlation with residual disease and survival outcomes was examined.

**Methods:**

A total of 286 AEOC patients (PDS: 174; NACT-IDS: 112) treated between 2011 and 2020 were retrospectively analyzed. Patients were stratified by Suidan score based on clinical and radiological criteria. Surgical outcomes, progression-free survival (PFS), and overall survival (OS) were compared between treatment groups across score categories.

**Results:**

In the PDS group, increasing Suidan scores correlated with higher rates of residual disease (RD), with 100% RD observed in patients scoring ≥6. NACT-IDS significantly reduced RD rates in patients with Suidan scores ≥2. Among high-score patients (≥6), NACT-IDS was associated with significantly prolonged PFS compared to PDS (27.2 vs. 16.3 months, P = 0.04), though OS remained comparable across groups.

**Conclusion:**

The Suidan score may be a useful preoperative tool for predicting surgical and progression outcomes in AEOC. Patients with high tumor burden (score ≥6) may benefit more from NACT-IDS, highlighting the importance of CT-based stratification in guiding personalized treatment strategies.

## 1 Introduction

Ovarian cancer ranks as the third most common gynecologic malignancy and is the leading cause of gynecologic cancer-related mortality globally (1), accounting for 3.7% of all cancer cases and 4.7% of cancer-related deaths in 2020 (2). Epithelial ovarian carcinoma (EOC) accounts for >90% of all malignant ovarian neoplasms (3). Due to the lack of specific clinical symptoms, most patients are diagnosed at advanced stages (FIGO III/IV), which significantly impacts treatment outcomes (4). The management of advanced-stage ovarian cancer typically involves two primary strategies: primary debulking surgery (PDS) and neoadjuvant chemotherapy followed by interval debulking surgery (NACT-IDS). In clinical practice, PDS is generally preferred when complete cytoreduction is considered achievable based on preoperative clinical and imaging assessment. Conversely, NACT-IDS is typically recommended for patients in whom optimal cytoreduction with primary surgery is unlikely to be feasible (5, 6).

Previous studies have shown similar outcomes for PDS and NACT-IDS in terms of survival. However, these studies have highlighted that NACT-IDS can significantly reduce rates of suboptimal tumor reduction, minimize residual disease, lower surgical complications, and shorten hospital stays (5, 7–10). Notably, Vergote et al. (11) combined data from 2010 and 2015, concluding that for patients with FIGO stage IV disease, NACT-IDS led to better overall survival (OS) and progression-free survival (PFS) compared to PDS (11). In the EORTC 55971 trial, the investigators found that patients with stage IIIC disease and metastatic tumors ≤45mm had improved survival rates when treated with PDS, whereas those with stage IV disease and metastatic tumors >45mm exhibited better survival outcomes following NACT-IDS (12).

Although NACT-IDS has gained increasing acceptance as primary treatment for AEOC, the determination of optimal initial therapeutic strategy continues to be a subject of ongoing clinical debate (13). Therefore, establishing an effective evaluation system to predict treatment efficacy and prognosis in patients with advanced epithelial ovarian cancer (AEOC) is of paramount clinical importance. Multiple evaluation modalities, including clinical parameters, serological biomarkers, genomic profiling, imaging characteristics, and laparoscopic assessments, have been developed to guide treatment selection in advanced ovarian cancer (14, 15). Biomarkers such as human epididymis protein 4(HE4), serum CA-125, and CA-153 demonstrate strong predictive capability for surgical outcomes (16–18). In addition, international consensus guidelines consistently recommend routine germline testing for pathogenic variants in BRCA1 and BRCA2, and other ovarian cancer susceptibility genes for all patients diagnosed with EOC (14). Identification of these molecular alterations enables healthcare systems to deliver personalized diagnostic, prognostic, and therapeutic strategies, while simultaneously providing critical risk assessment for family members (4, 6, 19). In clinical practice, composite scoring systems represent the most widely adopted strategy for surgical outcome prediction, including validated tools such as the Risk of Ovarian Malignancy Algorithm (ROMA) index (20), CT-based predictive models (21, 22), and laparoscopic scoring systems (23). In addition to CT-based predictive models, other imaging modalities such as PET/CT and MRI have also been investigated for evaluating tumor dissemination, predicting surgical resectability, and assessing prognosis in advanced ovarian cancer (24).

Among these, abdominal and pelvic enhanced CT scans are the most widely used imaging methods in the clinical management of ovarian cancer (13). The Suidan score, introduced for predicting surgical outcomes in ovarian cancer, is commonly employed in clinical practice (21). However, its application to predict the prognosis of patients with advanced ovarian cancer treated with PDS or NACT-IDS remains underexplored. The aim of this study was to preliminarily assess the prognostic value of the Suidan score in patients with AEOC.

## 2 Materials and methods

### 2.1 Population

This retrospective cohort study was approved by the Institutional Review Board of West China Second University Hospital, Sichuan University, with a waiver of informed consent. We retrospectively analyzed 528 patients with advanced epithelial ovarian cancer (AEOC) treated at our institution between 2011 and 2020. After excluding patients without serum CA-125 testing within 3 weeks before treatment, without contrast-enhanced abdominopelvic CT within 1 month before treatment, with concurrent secondary malignancies, or with incomplete clinical data, 286 patients were included in the final analysis. Chest CT was routinely performed for pre-treatment staging to assess thoracic metastases. Of these, 174 patients underwent primary debulking surgery (PDS) and 112 received neoadjuvant chemotherapy followed by interval debulking surgery (NACT-IDS). The initial treatment strategy (PDS vs. NACT-IDS) was determined by the multidisciplinary team on the basis of preoperative clinical assessment, imaging-estimated tumor burden, resectability, and the patient’s general condition and surgical tolerance. For patients undergoing NACT-IDS, the Suidan score was calculated exclusively based on baseline CT images obtained before initiation of chemotherapy. The cohort comprised histologically confirmed advanced-stage carcinomas originating from the ovary, fallopian tube, or peritoneum. Patients undergoing PDS received ≥4 cycles of platinum-based combination chemotherapy (carboplatin or cisplatin with paclitaxel) based on surgical-pathological evaluation. The NACT-IDS protocol consisted of 1–8 cycles of neoadjuvant platinum-taxane chemotherapy, with 2–4 cycles administered to 92.9% (104/112) of patients. Following IDS, additional platinum-based chemotherapy was administered for ≥4 cycles. Clinicopathological data including age, FIGO stage, CA-125 levels, ASA physical status, therapeutic regimen, and treatment initiation date were retrospectively collected from medical records. OS was defined as the time from diagnosis to death or the follow-up deadline of May 31, 2024. PFS was defined as the time from diagnosis to the date of disease progression or death from any cause. We used the date of diagnosis as the starting point to ensure a uniform time origin for both treatment groups in this retrospective cohort. Demographic and clinical characteristics of the study cohort are presented in **Table 1**.

**Table 1:** Demographic and clinical characteristics of patients.

Characteristic	PDS(n=174)	NACT+IDS(n=112)	P-value
Age(years)	50.0 ± 8.5	51.9 ± 9.8	0.103
Surgical outcome			<0.001
Complete cytoreduction (R0)	51(29.31%)	52(46.43%)	
Incomplete cytoreduction (non-R0)	123(70.69%)	60(53.57%)	
FIGO stage			0.040
IIIA	13(7.47%)	4(3.57%)	
IIIB	20(11.49%)	9(8.04%)	
IIIC	135(77.59%)	87(77.68%)	
IV	6(3.45%)	12(10.71%)	
Histology			0.088
Serous	154(88.51%)	107(95.54%)	
Mucinous	2(1.15%)	0(0.00%)	
Endometrioid	6(3.45%)	0(0.00%)	
Clear cell	4(2.30%)	0(0.00%)	
Mixed	8(4.59%)	5(4.46%)	
Grading			0.020
G1-2	19(10.92%)	3(2.68%)	
G3	155(89.08%)	109(97.32%)	
ASA class			0.234
1-2	153(87.93%)	92(82.14%)	
≥3	21(12.07%)	20(17.86%)	
CA-125			<0.001
<600U/mL	90(51.72%)	33(29.46%)	
≥600U/mL	84(48.28%)	79(70.54%)	

### 2.2 Stratification based on the Suidan CT scoring system

**Figure 1:**
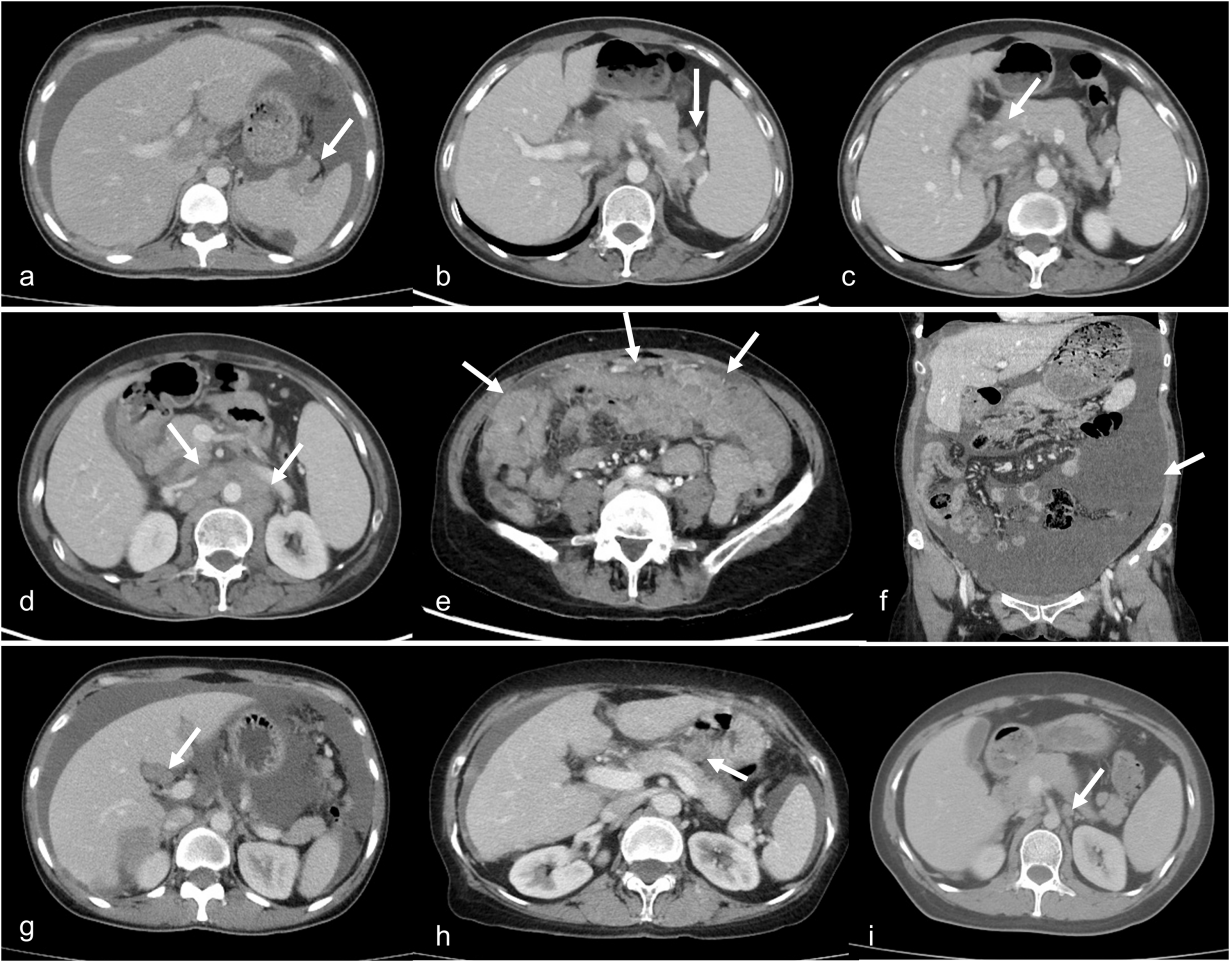
CT features assessed as part of the Suidan scoring system. Representative CT images demonstrating the radiologic parameters used in the Suidan score evaluation.**(a)** Lesion in splenic hilum/ligaments; **(b)** Lesion in splenic ligaments; **(c)** Porta hepatis lesion; **(d)** Retroperitoneal lymph nodes above the renal hilum; **(e)** Diffuse small bowel adhesions/thickening; **(f)** Abdominal ascites (moderate-severe); **(g)** Gallbladder fossa/Liver intersegmental fissure lesion; **(h)** Lesser sac lesion >1cm; **(i)** Root of the superior mesenteric artery lesion.

The CT-based scoring system used in this study was derived from the Suidan et al. (21) predictive model, incorporating three clinical parameters and eight radiologic features as detailed in **Table 2** (21). Representative radiologic manifestations corresponding to Suidan scoring criteria are presented in **Figure 1**. Three attending radiologists, blinded to both surgical outcomes and final histopathological findings, independently scored and stratified all patients in the PDS and NACT-IDS groups according to the Suidan scoring criteria. For cases with scoring discrepancies, a final consensus was achieved through structured discussion among all three radiologists. Within each treatment group (PDS/NACT-IDS), patients were stratified into four prognostic categories based on Suidan scores: low-risk (0–1), intermediate-low (2-3), intermediate-high (4-5), and high-risk (≥6). We comparatively analyzed survival outcomes between treatment strategies (PDS vs. NACT-IDS) across all Suidan score strata (0-1, 2-3, 4-5, and ≥6). We generated Kaplan-Meier survival curves to analyze PFS and OS, with between-group differences assessed by log-rank tests.

**Table 2:** Clinical and radiologic criteria of Suidan score.

Criteria	Predictive score
Age ≥60 years	1
CA-125 ≥600 U/mL	1
ASA ≥3	1
Lesion in splenic hilum/ligaments	1
Gastrohepatic ligament/Porta hepatis lesion	1
Retroperitoneal lymph nodes above the renal hilum (including supradiaphragmatic)	1
Diffuse small bowel adhesions/thickening	1
Abdominal ascites (moderate-severe)	2
Gallbladder fossa/Liver intersegmental fissure lesion	2
Lesser sac lesion >1 cm	2
Root of the superior mesenteric artery lesion	4

### 2.3 Statistical analysis

Statistical analyses were performed using SPSS 26.0 software. Continuous variables with normal distribution were presented as mean ± standard deviation (SD), analyzed using independent two-sample t-tests. Non-normally distributed data were expressed as median with interquartile range (IQR; Q1-Q3), compared via Mann-Whitney U tests. Categorical variables were evaluated using Pearson’s chi-square or Fisher’s exact tests, as appropriate. A two-tailed significance threshold of α=0.05 was applied throughout.

## 3 Results

### 3.1 Patient characteristics

A total of 286 patients with AEOC were included in the final analysis, comprising 174 patients treated with PDS and 112 with NACT-IDS. Baseline demographic and clinicopathologic characteristics are summarized in **Table 1**. No significant differences were observed in age or ASA status between groups (P>0.05). The NACT-IDS cohort exhibited a higher proportion of FIGO stage IV disease (10.71% vs. 3.45%, P = 0.040) and a significantly greater prevalence of elevated CA-125 levels ≥600 U/mL (70.54% vs. 48.28%, P<0.001). High-grade serous carcinoma predominated in both cohorts but was more frequent among patients receiving NACT-IDS (97.32% vs. 89.08%, P = 0.020).

### 3.2 Surgical outcomes according to Suidan score

Residual disease (RD), defined as any macroscopic tumor remaining after cytoreductive surgery (non-R0), demonstrated a clear and progressive association with increasing Suidan scores in the PDS group (**Table 3**). RD rates ranged from 54.43% in patients with scores 0–1 to 100% in those with scores ≥6. In contrast, patients receiving NACT-IDS experienced substantially improved cytoreductive outcomes across moderate-to-high score strata.

**Table 3:** Residual disease rates following PDS and NACT-IDS.

Suidan score	PDS RD rate (%)	NACT-IDS RD rate (%)	P-value
0-1	54.43%	54.55%	0.994
2-3	76.92%	51.52%	0.015
4-5	89.66%	47.22%	<0.001
≥6	100%	62.50%	0.09
total	70.69%	53.57%	0.03

For Suidan scores ≥2, RD rates were significantly lower in the NACT-IDS group than in the PDS group (all P ≤ 0.05). The most pronounced difference was observed among patients with scores 4–5, for whom RD rates decreased from 89.66% (PDS) to 47.22% (NACT-IDS) (P<0.001). Although high-score patients (≥6) continued to exhibit relatively elevated RD rates after NACT-IDS, the frequency of RD remained markedly lower than after PDS (62.50% vs. 100%, P = 0.09). Overall, the total RD rate was significantly reduced in the NACT-IDS cohort compared with PDS (53.57% vs. 70.69%, P = 0.03).

### 3.3 Progression-free survival

**Figure 2:**
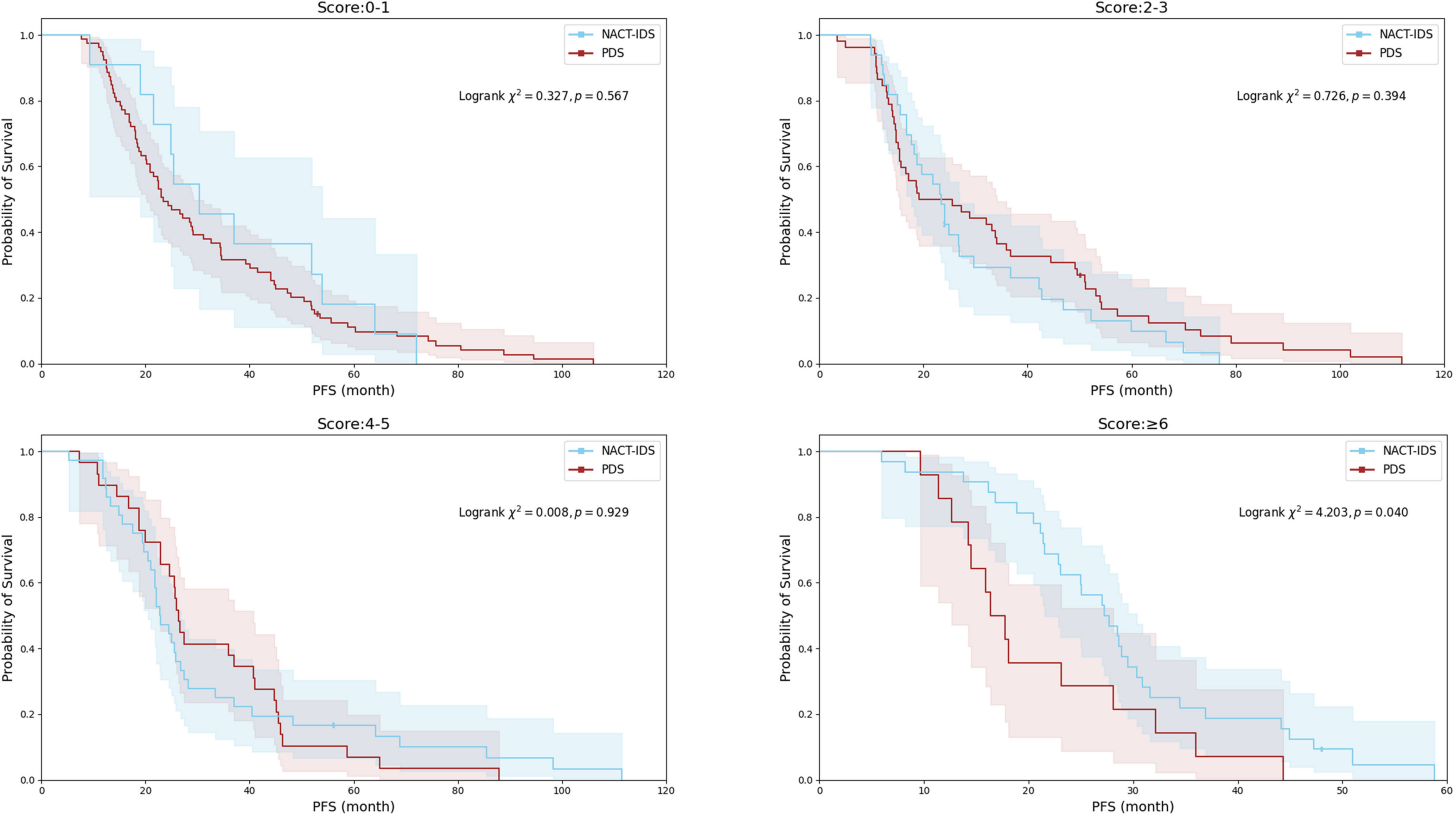
Kaplan-Meier analysis of PFS by Suidan score categories (0-1, 2-3, 4-5, ≥6) in PDS and NACT-IDS cohorts.

Kaplan–Meier analysis demonstrated comparable PFS between treatment strategies for patients with Suidan scores 0-1, 2-3, and 4-5 (all P>0.05) (**Figure 2**). However, a significant treatment-dependent divergence emerged in the high-risk subgroup (≥6 points). Patients with scores ≥6 who underwent NACT-IDS exhibited significantly prolonged PFS compared with those treated with PDS (median: 27.2 months [95% CI 22.4-32.0] vs. 16.3 months [95% CI 13.0-19.7], P = 0.040). This finding indicates that NACT-IDS confers a measurable oncologic advantage in patients with the highest preoperative tumor burden. .

### 3.4 Overall survival

**Figure 3:**
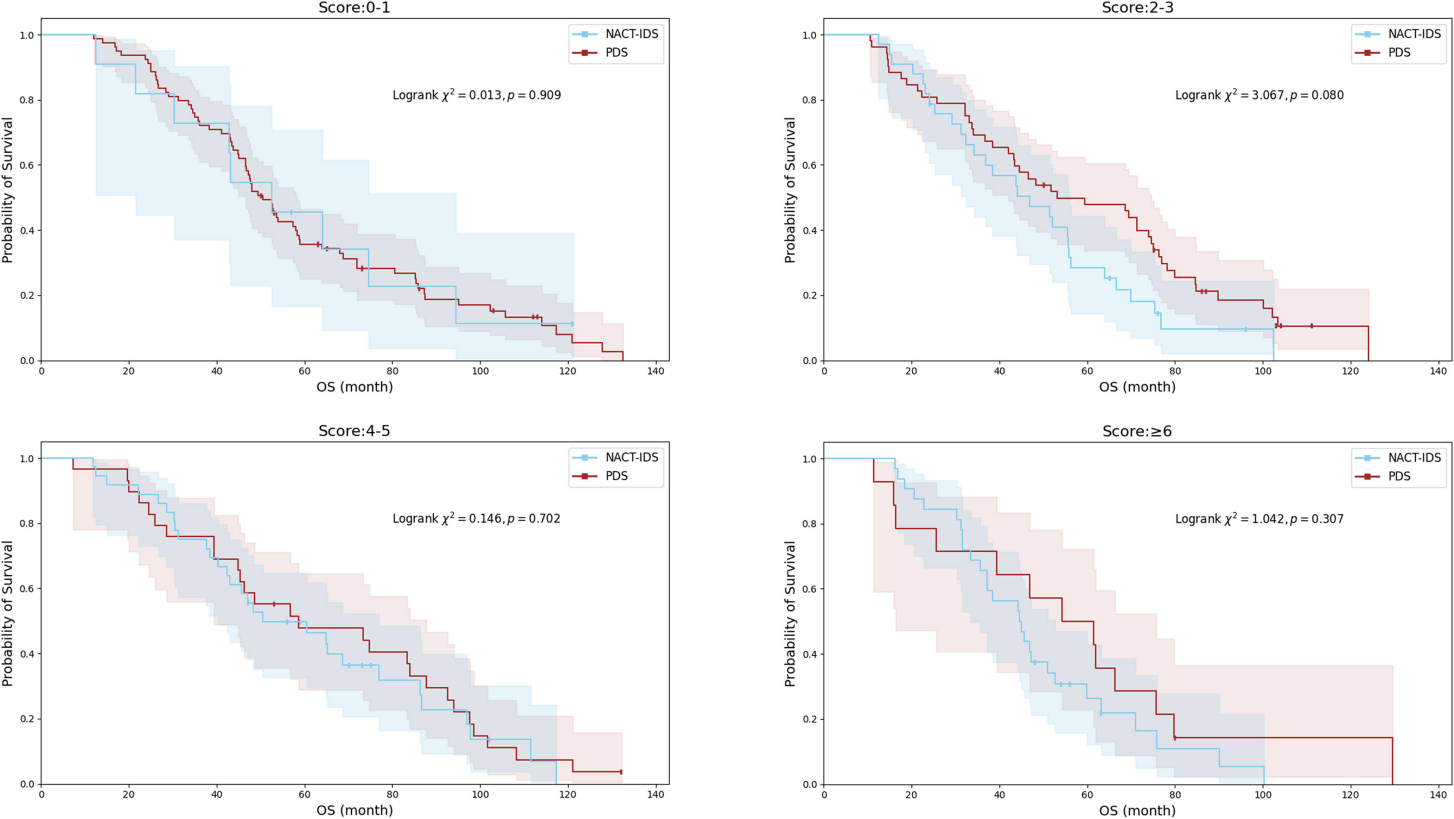
Kaplan-Meier analysis of OS stratified by Suidan score categories (0-1, 2-3, 4-5, ≥6) in NACT-IDS and PDS cohorts.

OS did not differ significantly between the PDS and NACT-IDS cohorts across all Suidan score categories (all P>0.05; **Figure 3**). For patients with scores 0-5, survival curves for both treatment strategies exhibited broad overlap, indicating comparable long-term outcomes.

Among patients with scores ≥6, the NACT-IDS cohort demonstrated a numerically shorter median OS compared with PDS (44.57 vs. 54.23 months), although this difference did not reach statistical significance (P = 0.307). A trend toward significance was observed in the 2–3 score group (P = 0.080), suggesting potential differential treatment effects that merit further exploration in larger prospective cohorts. Detailed comparisons of PFS and OS are shown in **Table 4**.

**Table 4:** Comparison of PFS and OS between NACT-IDS and PDS across Suidan score strata.

	Score	PDS		NACT-IDS		P
		Median	95% CI	Median	95% CI	
PFS	0-1	23.43	18.756-28.104	30.33	17.199-43.461	0.567
	2-3	19.1	5.202-32.998	23.4	18.448-28.352	0.394
	4-5	26.37	24.717-28.023	22.8	19.375-26.225	0.929
	≥6	16.33	12.975-19.685	27.2	22.391-32.009	0.04
OS	0-1	50.5	44.776-56.224	52.6	31.516-73.684	0.909
	2-3	53.1	26.899-79.301	46.93	29.099-64.761	0.080
	4-5	58.67	17.578-99.762	50.43	26.959-73.901	0.702
	≥6	54.23	27.352-81.108	44.57	34.453-54.687	0.307

## 4 Discussion

Contrast-enhanced abdominopelvic CT remains a cornerstone modality for preoperative assessment in ovarian cancer., owing to its noninvasive nature, widespread availability, and cost-effectiveness (13). To improve the prediction of surgical outcomes, several CT-based scoring systems that integrate radiologic and clinical parameters have been developed, including the Bristow model (25), the widely used Suidan scoring system (21, 26), and Peritoneal Cancer Index (PCI) (27). Historically, many of these models were designed to predict suboptimal cytoreduction, commonly defined as residual disease greater than 1 cm after surgery. However, contemporary clinical guidelines increasingly emphasize complete macroscopic cytoreduction (R0) as the most important surgical endpoint, as R0 resection is associated with the most favorable survival outcomes in advanced ovarian cancer. Beyond CT-based prediction models, other modalities have also been investigated for evaluating tumor dissemination and predicting surgical outcomes. Diffusion-weighted MRI has been explored as a complementary imaging technique for assessing peritoneal tumor burden and soft-tissue involvement because of its superior tissue contrast and functional characterization capabilities (28). Similarly, PET/CT can provide additional metabolic information and may assist in detecting occult metastatic disease and estimating the likelihood of complete cytoreduction in selected patients (29). Furthermore, laparoscopic evaluation remains an important adjunctive approach for estimating surgical feasibility. The laparoscopy-based predictive index proposed by Fagotti et al. demonstrated that structured intra-abdominal assessment can help identify patients unlikely to achieve optimal cytoreduction, thereby complementing imaging-based prediction models (22). Nevertheless, despite these advances, contrast-enhanced CT remains the most widely used modality in routine clinical practice because of its accessibility and its established role in preoperative staging and treatment planning.

The clinical utility of the Suidan score in predicting RD has been validated in an independent study (30), making it a practical tool in preoperative planning. However, the primary application of these models has been limited to surgical outcomes, with less emphasis on their ability to predict long-term prognostic endpoints. Although complete cytoreduction is a well-established independent prognostic factor (31), the overall prognosis of ovarian cancer is multifactorial, encompassing tumor biology, molecular features, and treatment responsiveness.

Among recognized prognostic indicators, residual disease following cytoreductive surgery is strongly associated with survival outcomes. Patients who achieve complete macroscopic resection (R0) have the most favorable survival outcomes. In contrast, patients with incomplete cytoreduction (non-R0) generally experience poorer prognosis. Therefore, contemporary clinical guidelines emphasize the distinction between complete and incomplete cytoreduction rather than the historical classification based on residual tumor size (32–34). In parallel, biomarkers such as preoperative and postoperative serum CA-125 levels are closely associated with tumor burden and survival (35, 36). Ascites, commonly observed in advanced-stage ovarian cancer-also serves as a significant indicator of disease progression and poor prognosis (37, 38). Given that the Suidan score incorporates CA-125, ASA physical status, and specific CT imaging features, it may serve not only as a surgical predictor but also as a surrogate marker for overall prognosis in AEOC.

Our findings reinforce the predictive strength of the Suidan score. A clear positive correlation was observed between increasing Suidan scores and the probability of residual lesions post-surgery. In the NACT-IDS group, 53.57% (60/112) of patients had incomplete cytoreduction (non-R0), compared with 70.69% (123/174) in the PDS group. Conversely, the rate of complete cytoreduction (R0) was higher in the NACT-IDS group than in the PDS group (46.43% vs. 29.31%). These results indicate that NACT-IDS is associated with superior cytoreductive outcomes, particularly in patients with extensive tumor spread—aligning with previous studies that favor NACT-IDS for improving surgical feasibility and reducing residual disease (5, 7–9). Incomplete cytoreduction in some patients may be attributed to extensive tumor dissemination involving surgically challenging anatomical locations, including the mesenteric root, porta hepatis, diffuse small bowel serosal involvement, and extensive diaphragmatic disease, which limit the feasibility of achieving complete tumor resection.

In the subgroup analysis stratified by Suidan scores, patients with scores ≥6 demonstrated significantly prolonged PFS in the NACT-IDS group compared to the PDS group (median PFS: 27.2 months, 95% CI 22.4-32.0 vs 16.3 months, 95% CI 13.0-19.7; P = 0.04). These findings suggest that the Suidan CT scoring system may provide useful information for predicting surgical outcomes and may offer additional prognostic insight in patients with advanced epithelial ovarian cancer.

It should be noted that the association observed between the Suidan score and survival outcomes may be partially mediated through surgical outcomes. Complete cytoreduction (R0) is a well-established independent prognostic factor in advanced ovarian cancer. Therefore, the prognostic value of the Suidan score observed in this study may reflect, at least in part, its ability to predict the likelihood of achieving complete tumor resection rather than representing a fully independent prognostic marker. These findings suggest that the Suidan score may have potential value in assisting treatment stratification, although further validation in prospective studies is required.

## 5 Conclusions

In conclusion, the Suidan score may represent a valuable preoperative imaging-based tool for guiding treatment selection in advanced ovarian cancer. Its prognostic value may help identify patients who could benefit most from a NACT-IDS approach and support individualized treatment planning. However, prospective multicenter studies are needed before routine clinical implementation can be recommended.

## 6 Limitations

Several limitations should be considered when interpreting the findings of this study. First, the retrospective design and lack of randomization may introduce selection bias and limit causal inference. Second, as all data were derived from a single tertiary center, the generalizability of the results to other institutions or populations may be limited. Third, although the Suidan scoring system has demonstrated strong predictive value for surgical outcomes, it was not originally developed to predict survival endpoints, and its prognostic implications therefore require further validation in prospective, multicenter studies. Additionally, treatment allocation between PDS and NACT-IDS was based on clinical judgment rather than randomization; patients with higher tumor burden or poorer surgical feasibility were more likely to receive NACT-IDS, which may introduce confounding when interpreting survival outcomes. Finally, important factors such as BRCA mutation status, treatment-related toxicity, and quality of life-key components of personalized oncology care-were not evaluated in this analysis and should be addressed in future studies.

## Data Availability

The raw data supporting the conclusions of this article will be made available by the authors, without undue reservation.
